# Supplementary material for: Stability of the marine nitrogen cycle over the past 165 million years
Source: Nat Commun. 2025 Oct 9;16:8982. doi: 10.1038/s41467-025-63604-x (PMC12511428; doi:10.1038/s41467-025-63604-x)
Supplement: Supplementary file 1 — Supplementary Information [file 41467_2025_63604_MOESM1_ESM.pdf]

Supplementary material for: Stability of the nitrogen cycle over the past 165 million years

Linda V. Godfrey,[1*] Anne Willem Omta,[2] Eli Tziperman,[3]
Xiang Li[4], Yongyun Hu[4], Paul G. Falkowski[1,5]

1 Department of Earth and Planetary Sciences, Rutgers University, Piscataway, NJ 08854, USA

2 Department of Earth, Environmental and Planetary Science, Case Western Reserve University, Cleveland, OH 44106, USA

3 Department of Earth and Planetary Sciences and School of Engineering and Applied Sciences, Harvard University, Cambridge, MA, 02138, USA

4 Laboratory for Climate and Ocean-Atmosphere Studies, Department of Atmospheric and Oceanic Sciences, School of Physics, Peking University, Beijing, China

5 Department of Marine and Coastal Sciences, Rutgers University, New Brunswick, NJ 08934, USA

***** Corresponding author. Email: linda.godfrey@rutgers.edu

Contents

1 Site descriptions **2**

2 Nitrogen cycle model **4**
- 2.1 Model description 4
- 2.2 Model equations 5
- 2.3 Fluxes 7
- 2.4 Why δ^{15}N depends nonlinearly and non-monotonously on mixing 11

3 Coastal upwelling calculation **12**

1 Site descriptions

We analyzed samples from a series of DSDP and ODP cores. They include sediment from sites 534, 402 and 137 located in Tethys and deposited between 165 and 90 million years ago, and site 762 on the northwest margin of Australia which was deposited from 90 million years ago to present. These site locations are near margins, but seaward of the shelf break, and chosen for their continuous and rapid accumulation to create favorable conditions for the preservation of δ^{15}N signals derived from particles (plankton and fecal matter) formed in overlying water and exported to the sediments. The other sites are 516 on the Rio Grande Rise deposited between 90 and 65 Myr ago, 689 and 690 on Maude Rise, deposited 65 to 24.4 Myr ago, and 608 in the north Atlantic deposited from 44 Myr ago to present. These cores are distant from the margins and sediment is carbonate rich. The N in these sites is derived from the export of particles in the overlying water, plankton, foraminifera, and fecal matter.

The oldest record comes from Site 534 off the Blake Spur and spans the period 165 to 122 Ma. The core consists of claystones and limestone, deposited in water which by the mid-Jurassic, was over 2500 m deep [1, 2]. Calcareous plankton indicate conditions of warm nutrient poor water punctuated by minor cooling at the end of the Jurassic. The opening of the gateway between western Tethys and the Panthalassic Ocean around 145 Ma established a circum-Equatorial current. Circulation models do not indicate proximity to upwelling (this study, [3]), the nearest site being off the north margin of S America.

Sites 402 and 137 span 121 to 94 Ma and 100 to 94 Ma respectively. Site 402 is located on the upper slope of the Bay of Biscay. The sampled section was deposited after conditons changed from small basins and tilted blocks to more open conditions with abundant planktonic foraminifera and coccoliths associated with either thermal subsidence or transgression. Black shale intervals (not sampled) indicate possible reworked shelf sediments [4]. Site 137 lies in the eastern N Atlantic basin and the section sampled was deposited on the ancestral mid-Atlantic Ridge. The core consists of hemipelagic clay and chalk ooze deposited above the CCD, under which oxygen contents were low [5]. Circulation models do not indicate coastal upwelling, rather suggesting the possibility of downwelling [3].

Site 762 spans 112 Ma to conclude the margin sites and $\delta^{15}\mathrm{N}_{\mathrm{DEEP}}$. It is located on the western part of the Exmouth Plateau [6] about 250 km off the coast of northwest Australia. From the mid Cretaceous onwards, sedimentation consists of hemipelagic and pelagic carbonate and show warmest temperatures at 115 Ma overprinted by shorter warmed and cooling periods [7]. Mid- to late-Cretaceous planktonic foraminifera assemblages indicate stable somewhat oligotrophic conditions punctuated by periods of thermocline disruption [8]. Over the latest Cretaceous, low diversity conditions switch to warm high diversity and increased Tethyan conditions [9]. Cyclic sedimentation occurred in the Late Cretaceous caused by orbital driven changes on land and recognized in the landward site cores, but no involvement of oxygen depleted bottom water was recognized [6]. Site 762 lies on the seaward side of the Exmouth Plateau Arch

and avoids focused bottom currents that created contourite channels or mass transport complexes [10]. Today, the shallow poleward Leeuwin current flows close to the margin, while the Leeuwin Undercurrent flows equatorward as an extension of the Western Australian Current (WAC), which is deflected west considerably south of the Exmouth Plateau. During the early- to mid-Cenozoic when Australia lay further south, the development of the Antarctic Circumpolar Current strengthened the WAC and may have lain at a latitude where upwelling might have been at the latitude around the Exmouth Plateau rather than its present position to the south (this work and [10]).

The oldest samples used to create the $\delta^{15}N_{DISTAL}$ record are from Site 516 on the Rio Grande Rise in 1300 m of water. The Rio Grande Rise is a steep sided guyot located 1300 km east of the edge of the S American shelf. Water over the top of the Rise was shallow around 83 Ma, the depth had increased to 1260 m by the Paleocene. The site has been remote from upwelling along the Walvis Ridge, and pelagic carbonate sedimentation records overall oligotrophic conditions [11].

Sites 689 and 690 are located on the Maud Rise in the Weddell Sea over 500km from the Antarctic coast. Maud Rise is an aseismic ridge which was at a depth well over 1000 m when the sediments we used were deposited [12]. The sites currently lie south of the Polar Front, but at the time of deposition, smectite dominated the clay fraction indicating much warmer conditions and a likely ice-free Antarctic continent [13]. Incomplete utilization of nitrate today leads to particulate and core top OM to have low $\delta^{15}N$ [14]. Partial consumption of nitrate is due to low Fe, low temperatures and growth rates, and water turnover rates at the surface that exceed biological demand due to upwelling and downwelling [15]. It is unclear whether these conditions would apply to the early Cenozoic.

Site 608 is located east of the Mid-Atlantic Ridge. The oldest sediments at the site are middle-late Eocene, when the site was on the ridge flank, but the site is now in more than 3500 m of water [16]. Pelagic sediments characterize the core and show that deep water was well-ventilated by 15 Ma through deepening of ridges to the north [16].

2 Nitrogen cycle model

2.1 Model description

To investigate which mechanisms could have given rise to the observed variations in the surface- and deep-ocean N, we use a multi-box biogeochemical model. The setup is based on a previous model for the marine nitrogen cycle [17], resolving N fixation, export production, nitrification, and denitrification. However, our new model setup explicitly includes isotopic fractionation between ^{14}N and ^{15}N (fractionation factors from [18]). The model has 3 compartments: a surface box, an intermediate-depth (Oxygen Minimum Zone) box through which water upwells, and a deep-ocean box. Water transports are prescribed, with a unidirectional overturning circulation and bidirectional diffusive exchanges between boxes. The bidirectional exchange between the surface and intermediate boxes is equal to 0. The associated transport rates and other parameter values are listed in Supplementary Table S1. Fig. 1 shows the schematic of the model. Throughout the following, subscripts s, i, r, and a will denote the surface, intermediate, rest of the ocean boxes, and atmosphere, respectively. All the stoichiometric ratios are given in Table 1, and all parameter values are in Table 2.

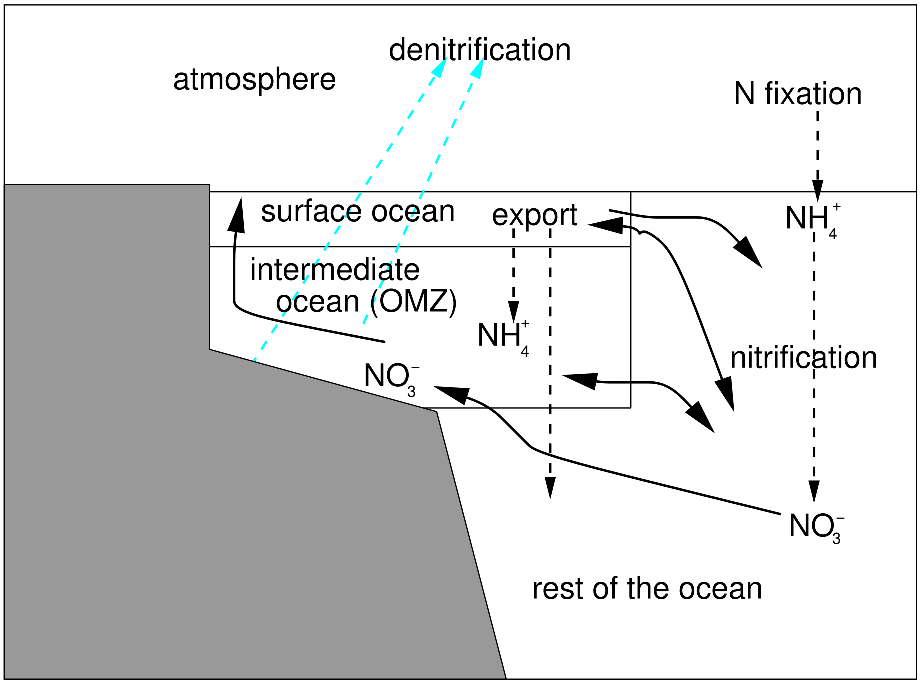

Figure 1: Schematic of the box model with the key N fluxes

2.2 Model equations

The equations for the surface box are:

$$
\begin{aligned}
\frac{d[\mathrm{NH}_4^+]_s}{dt} &= \frac{1}{V_s}\Big(- F_{N,ex}\frac{[\mathrm{NH}_4^+]_s}{N_s} + q\left([\mathrm{NH}_4^+]_i - [\mathrm{NH}_4^+]_s\right) \\
&\quad + q_{sr}\left([\mathrm{NH}_4^+]_r - [\mathrm{NH}_4^+]_s\right)\Big) \\
\frac{d[\mathrm{NO}_3^-]_s}{dt} &= \frac{1}{V_s}\Big(- F_{N,ex}\frac{[\mathrm{NO}_3^-]_s}{N_s} + q\left([\mathrm{NO}_3^-]_i - [\mathrm{NO}_3^-]_s\right) \\
&\quad + q_{sr}\left([\mathrm{NO}_3^-]_r - [\mathrm{NO}_3^-]_s\right)\Big) \\
\frac{d[^{15}\mathrm{NH}_4^+]_s}{dt} &= \frac{1}{V_s}\Big(- F_{^{15}N,ex,NH_4^+} + q\left([^{15}\mathrm{NH}_4^+]_i - [^{15}\mathrm{NH}_4^+]_s\right) \\
&\quad + q_{sr}\left([^{15}\mathrm{NH}_4^+]_r - [^{15}\mathrm{NH}_4^+]_s\right)\Big) \\
\frac{d[^{15}\mathrm{NO}_3^-]_s}{dt} &= \frac{1}{V_s}\Big(- F_{^{15}N,ex,NO_3^-} + q\left([^{15}\mathrm{NO}_3^-]_i - [^{15}\mathrm{NO}_3^-]_s\right) \\
&\quad + q_{sr}\left([^{15}\mathrm{NO}_3^-]_r - [^{15}\mathrm{NO}_3^-]_s\right)\Big) \\
\frac{d[\mathrm{PO}_4^{3-}]_s}{dt} &= \frac{1}{V_s}\Big(- \frac{F_{N,ex}}{R_{NP}} + q\left([\mathrm{PO}_4^{3-}]_i - [\mathrm{PO}_4^{3-}]_s\right) \\
&\quad + q_{sr}\left([\mathrm{PO}_4^{3-}]_r - [\mathrm{PO}_4^{3-}]_s\right)\Big)
\end{aligned}
$$

with $N_s \equiv [\mathrm{NH}_4^+]_s + [\mathrm{NO}_3^-]_s$ the surface DIN concentration, V_s the volume of the surface box, and transport rates q (upwelling) q_{xy} (bidirectional transport between boxes x and y). $F_{N,ex}$ is the total N flux from the surface box due to organic matter export; $F_{^{15}N,ex,NH_4^+}$ and $F_{^{15}N,ex,NO_3^-}$ indicate the 15N fluxes in export production fueled by NH_4^+ and NO_3^-, respectvely. These and other fluxes are defined in Section 2.3. We set $[\mathrm{NH}_4^+]_r$ to zero assuming all ammonium in the rest of the ocean is instantaneously nitrified, which means that $N_r = [\mathrm{NO}_3^-]_r$. There is no dynamical equation for the oxygen concentration at the ocean surface ($[\mathrm{O}_2]_s$), because it is assumed to be constant (set by the atmospheric oxygen concentration and the temperature).

The following equations describe the intermediate box:

$$\frac{d[\mathrm{NH}_4^+]_i}{dt} = \frac{1}{V_i}\Big(F_{N,ex} - F_{N,bur} - F_{N,nit} + (q + q_{ir})\left([\mathrm{NH}_4^+]_r - [\mathrm{NH}_4^+]_i\right)\Big)$$

$$\frac{d[\mathrm{NO}_3^-]_i}{dt} = \frac{1}{V_i}\Big(F_{N,nit} - R_{den}\left(F_{N,den} + F_{N,den\_sed}\right) + (q + q_{ir})\left([\mathrm{NO}_3^-]_r - [\mathrm{NO}_3^-]_i\right)\Big)$$

$$\frac{d[^{15}\mathrm{NH}_4^+]_i}{dt} = \frac{1}{V_i}\Big(F_{^{15}N,ex} - F_{^{15}N,bur} - F_{^{15}N,nit} + (q + q_{ir})\left([^{15}\mathrm{NH}_4^+]_r - [^{15}\mathrm{NH}_4^+]_i\right)\Big)$$

$$\frac{d[^{15}\mathrm{NO}_3^-]_i}{dt} = \frac{1}{V_i}\Big(F_{^{15}N,nit} - R_{den}\left(F_{^{15}N,den} + F_{^{15}N,den\_sed}\right) + (q + q_{ir})\left([^{15}\mathrm{NO}_3^-]_r - [^{15}\mathrm{NO}_3^-]_i\right)\Big)$$

$$\frac{d[\mathrm{PO}_4^{3-}]_i}{dt} = \frac{1}{V_i}\Big(\frac{F_{N,ex} - F_{N,bur}}{R_{NP}} + F_{P,rer} + (q + q_{ir})\left([\mathrm{PO}_4^{3-}]_r - [\mathrm{PO}_4^{3-}]_i\right)\Big)$$

$$\frac{d[\mathrm{O}_2]_i}{dt} = \frac{1}{V_i}\left(-F_{N,aer}R_{ON} - 2F_{N,nit} + (q + q_{ir})\left([\mathrm{O}_2]_r - [\mathrm{O}_2]_i\right)\right)$$

We take the stoichiometric factor R_{ON} (the O_2:N ratio of aerobic remineralization of organic matter) equal to the standard C:N ratio of oceanic organic matter, because 1 mol O_2 is consumed per mol of organic C that is oxidized through aerobic metabolism. The stoichiometric factor R_{den} (the number of mol NO_3^- lost per N-mol organic matter oxidized through denitrification) equals 0.8*R_{ON}, because 1 mol O_2 can accept 4 mol electrons whereas 1 mol NO_3^- can accept 5 mol electrons.

As we neglect NH_4^+ in the rest of the ocean, we have:

$$\frac{dN_r}{dt} = \frac{1}{V_r}\Big(F_{N,fix} + (q + q_{sr})\left(N_s - N_r\right) + q_{ir}\left(N_i - N_r\right)\Big)$$

$$\frac{d^{15}N_r}{dt} = \frac{1}{V_r}\Big(F_{^{15}N,fix} + (q + q_{sr})\left(^{15}N_s - {}^{15}N_r\right) + q_{ir}\left(^{15}N_i - {}^{15}N_r\right)\Big)$$

$$\frac{d[\mathrm{PO}_4^{3-}]_r}{dt} = \frac{1}{V_r}\Big(F_{P,wea} + (q + q_{sr})\left([\mathrm{PO}_4^{3-}]_s - [\mathrm{PO}_4^{3-}]_r\right) + q_{ir}\left([\mathrm{PO}_4^{3-}]_i - [\mathrm{PO}_4^{3-}]_r\right)\Big)$$

We calculate the oxygen concentration in the rest of the ocean box based on the stoichiometry of oxygen consumption, accounting for aerobic oxidation of organic matter and nitrification:

$$[\mathrm{O}_2]_r = [\mathrm{O}_2]_s \underbrace{-R_{ON}N_r}_{\text{Oxidation}} \underbrace{-2N_r}_{\text{Nitrification}}$$

2.3 Fluxes

The export production flux ($F_{N,ex}$) can be limited either by DIN (NH_4^+/NO_3^-) or PO_4^{3-}, according to Liebig's law of the minimum:

$$F_{N,ex} = k_{ex} \min\left(N_s, R_{NP}[PO_4^{3-}]_s\right)$$

with rate constant k_{ex} and R_{NP} the N:P ratio of the exported organic matter. NH_4^+ and NO_3^- are exported in the same ratio in which they occur in the surface box, i.e., the primary producers do not have a preference for either form of DIN. NH_4^+ utilization in export production involves stronger isotopic fractionation (ε_{ex,NH_4^+}=15‰) than utilization of NO_3^- (ε_{ex,NO_3^-}=5‰). Some of the organic matter is denitrified in the sediments, according to a Michaelis-Menten dependence on the NO_3^- in the intermediate box without isotopic fractionation:

$$F_{N,den\_sed} = F_{N,ex} S_{den\_sed} \frac{[NO_3^-]_i}{[NO_3^-]_i + K_{den}}$$

with S_{den_sed} the maximum fraction of the export being denitrified in the sediments and half-saturation constant K_{denit}. The remaining organic matter is exported to the intermediate box ($F_{N,ex,i}$).

Oxidation of organic matter in the intermediate box takes place preferentially aerobically, according to a Michaelis-Menten dependence on O_2:

$$F_{N,aer} = F_{N,ex,i} \frac{[O_2]_i}{[O_2]_i + K_{ox}}$$

with half-saturation constant K_{ox}. This formulation implies that at high O_2 concentrations, all exported organic matter is oxidized through sediment denitrification and aerobic metabolism. At low O_2 concentrations, a fraction of the remaining exported organic matter is oxidized through denitrification in the water column of the intermediate box, according to a Michaelis-Menten dependence on NO_3^- and isotopic fractionation with $\varepsilon_{den} = 25$‰:

$$F_{N,den} = (F_{N,ex,i} - F_{N,aer}) \frac{[NO_3^-]_i}{[NO_3^-]_i + K_{den}}$$

The organic matter that is not oxidized either aerobically or through denitrification is buried:

$$F_{N,bur} = F_{N,ex,i} - F_{N,aer} - F_{N,den}$$

There exists substantial empirical evidence for phosphorus re-release from sediments back into the water column under low-oxygen conditions [19, 20, 21]. We represent this process through a phosphorus re-released flux ($F_{P,rer}$):

$$F_{P,rer} = \frac{F_{N,bur}}{R_{NP}} S_{P,rer} \left(1 - \frac{[O_2]_i}{[O_2]_i + K_{ox}}\right)$$

with $S_{P,rer}$ the maximum re-released fraction.

Nitrification converts NH_4^+ into NO_3^- and can be limited by either NH_4^+ or O_2, according to a Liebig minimum formulation:

$$F_{N,nit} = k_{nit} \min\left([\mathrm{NH}_4^+]_i, 2[\mathrm{O}_2]_i\right)$$

with rate constant k_{nit}; the stoichiometric factor 2 reflects that 2 mol O_2 is needed to nitrify 1 mol NH_4^+. The nitrification process involves isotopic fractionation (ε_{nit}=25‰).

N fixation adds N from the atmosphere to the rest of the ocean box. The N fixation flux ($F_{N,fix}$ in mol/s) depends on the deficit of DIN with respect to P in the rest of the ocean box, according to a sigmoid function:

$$F_{N,fix} = F_{N,fix,m}\left(0.5 + \frac{1}{\pi}\arctan\left(\frac{-\pi N_r^*}{N_0^*}\right)\right)$$

with $F_{N,fix,m}$ the maximum N fixation flux. As $N_r^* \equiv N_r - 16[\mathrm{PO}_4^{3-}]_r$ [22, 23], a deficit of N vs P leads to N fixation, whereas deficits of P vs N suppress N fixation. We take N_0^* equal to 1 nM, so that deficits of N or P at the surface will generally remain within the nM range. The atmosphere is assumed to be an infinite reservoir with δ^{15}N of 0‰; no isotopic fractionation takes place during N fixation. There is a weathering input of P to the rest of the ocean box ($F_{P,wea}$) that is varied in the simulations.

In the equations below, $R_X^{15} = {}^{15}X/({}^{15}X + {}^{14}X)$ indicates the isotopic mixing ratio in a compartment, where X can denote $[\mathrm{NH}_4^+]_s$ or $[\mathrm{NO}_3^-]_d$, etc.

$$\begin{aligned}
F_{^{15}N,ex,NH_4^+} &= F_{N,ex}\frac{[\mathrm{NH}_4^+]_s}{N_s}\frac{R^{15}_{[\mathrm{NH}_4^+]_s}\left(1-10^{-3}\varepsilon_{ex,\mathrm{NH}_4^+}\right)}{1-R^{15}_{[\mathrm{NH}_4^+]_s}10^{-3}\varepsilon_{ex,\mathrm{NH}_4^+}}\\
F_{^{15}N,ex,NO_3^-} &= F_{N,ex}\frac{[\mathrm{NO}_3^-]_s}{N_s}\frac{R^{15}_{[\mathrm{NO}_3^-]_s}\left(1-10^{-3}\varepsilon_{ex,\mathrm{NO}_3^-}\right)}{1-R^{15}_{[\mathrm{NO}_3^-]_s}10^{-3}\varepsilon_{ex,\mathrm{NO}_3^-}}\\
F_{^{15}N,ex} &= F_{^{15}N,ex,NH_4^+} + F_{^{15}N,ex,NO_3^-}\\
F_{^{15}N,nit} &= F_{N,nit}\frac{R^{15}_{[\mathrm{NH}_4^+]_i}\left(1-\varepsilon_{nit}\right)}{1-R^{15}_{[\mathrm{NH}_4^+]_i}10^{-3}\varepsilon_{nit}}\\
F_{^{15}N,den} &= F_{N,den}\frac{R^{15}_{[\mathrm{NO}_3^-]_i}\left(1-10^{-3}\varepsilon_{den}\right)}{1-R^{15}_{[\mathrm{NO}_3^-]_i}10^{-3}\varepsilon_{den}}\\
F_{^{15}N,den_sed} &= F_{N,den_sed}R^{15}_{[\mathrm{NO}_3^-]_i}\\
F_{^{15}N,fix} &= F_{N,fix}R^{15}_{\mathrm{N}_{2,a}}
\end{aligned}$$

Table 1: Definitions and values of stoichiometric ratios used in the equations

Ratio	Definition	Value
$R^{15}_{\mathrm{N}_{2,a}}$	$\frac{p_{^{15}\mathrm{N}_{2,a}}}{p_{\mathrm{N}_{2,a}}}$	0.00366353
$R^{15}_{[\mathrm{NH}_4^+]_s}$	$\frac{[^{15}\mathrm{NH}_4^+]_s}{[\mathrm{NH}_4^+]_s}$	Variable
$R^{15}_{[\mathrm{NH}_4^+]_i}$	$\frac{[^{15}\mathrm{NH}_4^+]_i}{[\mathrm{NH}_4^+]_i}$	Variable
$R^{15}_{[\mathrm{NH}_4^+]_d}$	$\frac{[^{15}\mathrm{NH}_4^+]_d}{[\mathrm{NH}_4^+]_d}$	Variable
$R^{15}_{[\mathrm{NO}_3^-]_s}$	$\frac{[^{15}\mathrm{NO}_3^-]_s}{[\mathrm{NO}_3^-]_s}$	Variable
$R^{15}_{[\mathrm{NO}_3^-]_i}$	$\frac{[^{15}\mathrm{NO}_3^-]_i}{[\mathrm{NO}_3^-]_i}$	Variable
$R^{15}_{[\mathrm{NO}_3^-]_d}$	$\frac{[^{15}\mathrm{NO}_3^-]_d}{[\mathrm{NO}_3^-]_d}$	Variable
R_{NP}	N:P ratio of export	20
R_{ON}	O_2:N ratio of aerobic respiration	6.6
R_{den}	mol NO_3^- loss per N-mol organic matter oxidized through denitrification	5.28 (=0.8*R_{ON})

Table 2: Description of multi-box model parameters (par), with their respective units, interpretations and values for the standard case simulation.

Par	Units	Interpretation	Standard value
V_s	m^3	Surface box volume	10^{13}
V_i	m^3	Intermediate box volume	10^{14}
V_r	m^3	Rest of ocean volume	$1.51*10^{18}$
q	Sv	Upwelling transport	0.1
q_{sr}	Sv	Bidirectional transport between surface and rest of ocean boxes	0.1
q_{ir}	Sv	Bidirectional transport between intermediate and rest boxes	0.2
$F_{N,fix,m}$	kmol/s	Maximum N-fixation flux	20
k_{ex}	s^{-1}	N export rate	$1.0*10^{-6}$
S_{den_sed}	–	Max fraction of export denitrified in sediments	0.03
S_e	–	Max fraction of export into intermediate box	0.25
S_{den}	–	Max fraction of export into intermediate box denitrified in water column	0.1
$S_{P,rer}$	–	Max fraction of buried P re-released into ocean	0.5
$[O_2]_s$	mM	Surface O_2 concentration	0.35
K_{ox}	nM	Saturation constant of aerobic respiration	10
K_{den}	nM	Saturation constant of denitrification	10
N_0^*	nM	N/P deficit scaling parameter	1.0
ε_{ex,NH_4^+}	‰	NH_4^+ export fractionation	15
ε_{ex,NO_3^-}	‰	NO_3^- export fractionation	5
ε_{nit}	‰	Nitrification fractionation	25
ε_{den}	‰	Water-column denitrification fractionation	25

2.4 Why δ^{15}N depends nonlinearly and non-monotonously on mixing

In this Section, we will focus on $\delta^{15}N_r$, but essentially the same argument holds for the other boxes. $\delta^{15}N_r$ is proportional to $\frac{^{15}N_r}{N_r}$. Using that $\frac{dN_r}{dt} = \frac{d^{15}N_r}{dt} = 0$ at steady state and that $N_s \approx 0$, we obtain:

$$\frac{^{15}N_r}{N_r} \approx \frac{F_{^{15}N,fix} + q_{ir}\,^{15}N_i}{F_{N,fix} + q_{ir}N_i} \tag{1}$$

which is a nonlinear equation. Using that $F_{^{15}N,fix} = F_{N,fix}R^{15}_{\mathrm{N}_{2,a}}$, we can rewrite equation (1) as:

$$\begin{aligned}\frac{^{15}N_r}{N_r} &\approx \frac{F_{N,fix}R^{15}_{\mathrm{N}_{2,a}} + q_{ir}N_i\frac{^{15}N_i}{N_i}}{F_{N,fix} + q_{ir}N_i} \\ &= R^{15}_{\mathrm{N}_{2,a}} + \frac{q_{ir}N_i\left(\frac{^{15}N_i}{N_i} - R^{15}_{\mathrm{N}_{2,a}}\right)}{F_{N,fix} + q_{ir}N_i} \\ &= R^{15}_{\mathrm{N}_{2,a}} + \frac{\frac{^{15}N_i}{N_i} - R^{15}_{\mathrm{N}_{2,a}}}{\frac{F_{N,fix}}{q_{ir}N_i} + 1} \end{aligned} \tag{2}$$

$R^{15}_{\mathrm{N}_{2,a}}$ is constant, but $F_{N,fix}$ and $\frac{^{15}N_i}{N_i}$ increase with mixing, while N_i decreases with mixing. This then explains the non-monotonous behavior.

3 Coastal upwelling calculation

We use the model results of [24] to estimate the total coastal upwelling mass flux every 10 Myr over the past 260 Myr. The local upwelling flux is given by,

$$M = \frac{\tau_{ac}}{f}. \tag{3}$$

Our first step in calculating the global integral of this flux is finding the direction of the coastline by fitting the local coastal outline at each location using the land mask in 3 nearby grid points. Every coastal point which we denote (x_2, y_2) where the coordinates are in degree longitude and latitude has two neighbors that are used to calculate the direction of the coast there. let these neighbors be (x_1, y_1), and (x_3, y_3). We would like to fit a parametric curve to the three points,

$$x = a_x t^2 + b_x t + c_x$$
$$y = a_y t^2 + b_y t + c_y.$$

We, therefore, need to calculate 6 coefficients. For this purpose, we let $t = 0$ at (x_1, y_1), $t = 0.5$ at (x_2, y_2), $t = 1$ at (x_3, y_3). So when evaluated at all 3 points, we have 6 equations for the six unknowns $a_x, b_x, c_x, a_y, b_y, c_y$.

The fit is done using NCL function `lspoly`, which calculates a set of coefficients for a weighted least squares polynomial fit to the given data. It is necessary that the number of data points be greater than n (the number of coefficients).

Given the fit, the tangent vectors are calculated as

$$\hat{\mathbf{s}}_{x_2,y_2} = (s_x, s_y) = \left(\frac{dx}{dt}, \frac{dy}{dt}\right)$$

after normalization to a unit length, so that $\hat{\mathbf{s}}$ is now a unit vector. This tangent vector needs to be translated to units of m rather than degrees before being used to calculate the along-shore wind stress so that the final tangent vectors are given by

$$\hat{\mathbf{t}}_{x_2,y_2} = (s_x R \cos\theta, s_y R),$$

and then $\hat{\mathbf{t}}_{x_2,y_2}$ also needs to be normalized. The coastal length is then given by

$$\int_{0.25}^{0.75} dt,$$

and the coastal upwelling transport is finally given by

$$M = \sum_i \int_{0.25}^{0.75} dt \;\max\left(0, \frac{\hat{\mathbf{t}}_{x_2,y_2}\vec{\tau}_{ac}}{f}\right),$$

where the sum is over all coastal grid points.

Next, we find the unit tangent vector to the coastal curve by taking the derivative of the fitted curve on the center coastal ocean grid we find the tangential equation. The direction of the unit tangent vector is determined as follows. 1) Write a normal vector A on the center coastal ocean grid, no matter its length or direction. 2) Find the nearest land grid that is adjacent to the center coastal ocean grid, and write a vector B that is pointing from the land point to the center coastal ocean grid. 3) Do a dot product between A and B. If it is positive (negative), the direction of A is pointing out of (into) the continent. In this way, we are able to determine the direction of the normal vector and thus can determine the unit tangent vector that goes counterclockwise along the continent.

The wind stress projected on the unit tangent vector is then

$$\tau_{ac} = \vec{\tau} \cdot \hat{t} \tag{4}$$

Finally, we calculate the upwelling flux as

$$M = \max(0, \frac{\tau_{ac}}{f}), \tag{5}$$

and integrate it globally. The results for the upwelling flux, upwelling flux using present-day continents, and using present-day winds are shown in Fig. 2, which also shows the evolution of the coastal length during the modeled period.

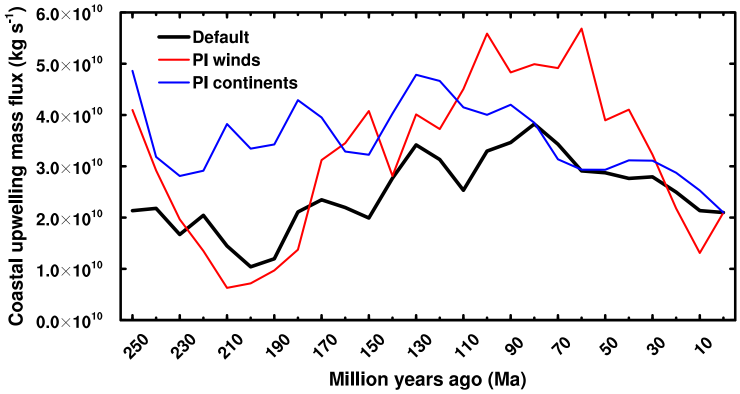

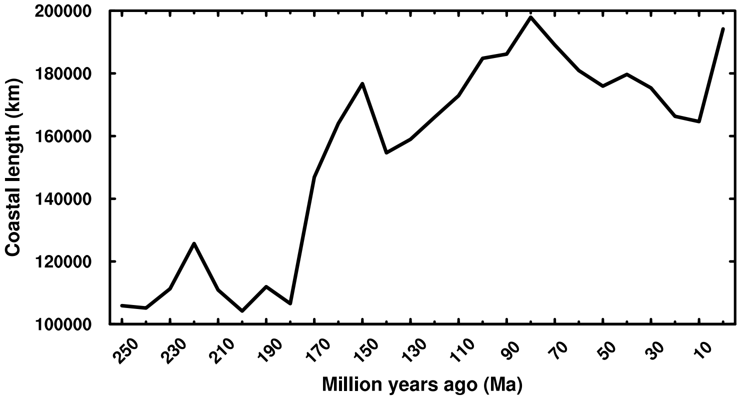

Figure 2: Upper: Evolution of coastal upwelling mass flux. Lower: Evolution of coastal length

References

[1] R. E. Sheridan, F. M. Gradstein, L. A. Barnard, D. M. Bliefnick, D. Habib, P. D. Jenden, H. Kagami, E. Keenan, J. Kostecki, K. A. Kvenvolden, M. Moullade, J. Ogg, A. H. F. Robertson, P. Roth, and T. H. Shipley. *Initial Reports of the Deep-Sea Drilling Project, Volume 76.* US Government Printing Office, Washington DC, 1983.

[2] F. Tremolada, A. Bornemann, T. J. Bralower, C. Koebarl, and B. van de Schootbrugge. Paleoceanographic changes across the Jurassic/Cretaceous boundary: The calcareous phytoplankton response. *Earth and Planetary Science Letters*, 241:361–371, 2006.

[3] R. P. M. Topper, J. Trabucho Alexandre, E. Tuenter, and P. T. Meijer. A regional ocean circulation model for the mid-Cretaceous North Atlantic Basin: Implications for black shale formation. *Climate of the Past*, 7:277–297, 2011.

[4] L. Montadert, D. G. Roberts, G. A. Auffret, W. D. Bock, P. A. Dupeuble, E. A. Hailwood, W. E. Harrison, H. Kagami, D. N. Lumsden, C. M. Muller, D. Schnitker, R. W. Thompson, T. L. Thompson, and P. P. Timofeev. *Initial Reports of the Deep-Sea Drilling Project, Volume 48.* US Government Printing Office, Wasington DC, 1979.

[5] D. E. Hayes, A. C. Pimm, J. P. Beckmann, W. E. Benson, W. H. Berger, P. H. Roth, P. R. Supko, and U. von Rad. *Initial Reports of the Deep-Sea Drilling Project, Volume 14.* US Government Printing Office, Washington DC, 1970.

[6] B. Ul-Haq, U. von Rad, S. O'Connell, A. Bent, C. D. Blome, P. E. Borella, R. Boyd, T. J. Bralower, W. W. Brenner, E. H. de Carlo, T. Dumont, N. Exon, B. Galbrun, X. Golovchenko, N. Görür, M. Ito, J. M. Lorenzo, P. A. Meyers, I. Moxon, D. K. O'Brien, M. Oda, M. Sart, W. G. Siesser, L. R. Snowdon, C. Tang, R. H. Wilkens, P. Williamson, and A. A. H. Wonders. *Proceedings of the Ocean Drilling Program, Volume 122.* US Government Printing Office, Washington DC, 1983.

[7] L. J. Clarke and H. C. Jenkyns. New oxygen isotope evidence for long-term Cretaceous climatic change in the Southern Hemisphere. *Geology*, 27:699–702, 1999.

[8] M. R. Petrizzo. Palaeoceanographic and palaeoclimatic inferences from Late Cretaceous planktonic foraminiferal assemblages from the Exmouth Plateau (ODP Sites 762 and 763, Eastern Indian Ocean). *Marine Micropaleonotology*, 45:117–150, 2002.

[9] M. A. Zepeda. Planktonic foraminiferal diversity, equitability and biostratigraphy of the uppermost Campanian-Maastrichtian, ODP Leg 122,

Hole 762C, Exmouth Plateau, NW Australia, Eastern Indian Ocean. *Cretaceous Research*, 19:117–152, 1998.

[10] H. D. Nugraha, C. A. L. Jackson, H. D. Johnson, D. M. Hodgson, and M. T. Reeve. Tectonic and oceanographic process interactions archived in Late Cretaceous to Present deep-marine stratigraphy on the Exmouth Plateau, offshore NW Australia. *Basin Research*, 31:405–430, 2019.

[11] J. G. V. Widmark. Biogeography of terminal Cretaceous benthic foraminifera: Deep-water circulation and trophic gradients in the deep South Atlantic. *Cretaceous Research I*, 21:367–379, 2000.

[12] J. P. Kennett and L. D. Stott. Abrupt deep-sea warming, palaeoceanographic changes and benthic extinctions at the end of the Palaeocene. *Nature*, 353:225–229, 1991.

[13] P. F. Barker, J. P. Kennett, S. O'Connell, S. Berkowitz, W. R. Bryant, L. H. Burckle, P. K. Egeberg, D. K. Fütterer, R. E. Gersonde, X. Golovchenko, N. Hamilton, L. Lawver, D. B. Lazarus, M. Lonsdale, B. Mohr, T. Nagao, C. P. G. Pereira, C. J. Pudsey, C. M. Robert, E. Schandl, V. Spiess, L. D. Stott, E. Thomas, K. F. M. Thompson, and S. W. Wise. *Proceedings of the Ocean Drilling Program, Volume 113*. US Government Printing Office, Washington DC, 1988.

[14] M. A. Altabet and R. François. Sedimentary nitrogen isotopic ratio as a recorder for surface ocean nitrate utilization. *Global Biogeochemical Cycles*, 8:103–116, 1994.

[15] F. Fripiat, A. Martínez-García, S. E. Fawcett, P. C. Kemeny, A. S. Studer, S. M. Smart, F. Rubach, S. Oleynik, D. M. Sigman, and G. H. Haug. The isotope effect of nitrate assimilation in the Antarctic Zone: Improved estimates and paleoceanographic implications. *Geochimica and Cosmochimica Acta*, 247:261–279, 2019.

[16] W. F. Ruddiman, R. B. Kidd, J. G. Baldauf, B. M. Clement, J. F. Dolan, M. R. Eggers, P. R. Hill, L. D. Keigwin, M. Mitchell, I. Philipps, F. Robinson, S. A. Salehipour, T. Takayama, E. Thomas, G. Unsold, and P. P. E. Weaver. *Initial Reports of the Deep-Sea Drilling Project, Volume 94*. US Government Printing Office, Wasington DC, 1987.

[17] K. Fennel, M. J. Follows, and P. G. Falkowski. The co-evolution of the nitrogen, carbon and oxygen cycles in the Proterozoic ocean. *American Journal of Science*, 305:526–545, 2005.

[18] D. M. Sigman and K. L. Casciotti. Nitrogen isotopes in the ocean. In J. H. Steele, K. K. Turekian, and S. A. Thorpe, editors, *Encyclopedia of Ocean Sciences*, pages 1884–1894, London, 2001. Academic Press.

[19] E. D. Ingall and R. Jahnke. Evidence for enhanced phosphorus regenration from marine sedfiments overlain by oxygen depleted waters. *Geochimica et Cosmochimica Acta*, 58:2571–2575, 1994.

[20] I. Tsandev, D. C. Reed, and C. P. Slomp. Phosphorus diagenesis in deep-sea sediments: Sensitivity to water column conditions and global scale implications. *Chemical Geology*, 330-331:127–139, 2012.

[21] F. Sulu-Gambari, M. Hagens, T. Behrends, D. Seitaj, F. J. R. Meysman, J. Middelburg, and C. P. Slomp. Phosphorus cycling and burial in sediments of a seasonally hypoxic marine basin. *Estuaries and Coasts*, 41:921–939, 2018.

[22] N. Gruber and J. L. Sarmiento. Global patterns of marine nitrogen fixation and denitrification. *Global Biogeochemical Cycles*, 11:809–837, 1997.

[23] C. Deutsch and T. Weber. Nutrient ratios as a tracer and driver of ocean biogeochemistry. *Annual Review of Marine Science*, 4:113–141, 2012.

[24] X. Li, Y. Hu, J. Guo, J. Lan, Q. Lin, X. Bao, S. Yuan, M. Wei, Z. Li, K. Man, Z. Yin, J. Han, J. Zhang, C. Zhu, Z. Zhao, Y. Liu, J. Yang, and J. Nie. A high-resolution climate simulation dataset for the past 540 million years. *Scientific Data*, 9:371, 2022.
